# Validation of Quantitative Ultrasonography for Osteoporosis Diagnosis in Postmenopausal Women Compared to Dual-Energy X-Ray Absorptiometry (DEXA)

**DOI:** 10.7759/cureus.38562

**Published:** 2023-05-04

**Authors:** Narendra S Kushwaha, Arpit Singh, Sanjiv Kumar, Dharmendra Kumar, Abhinav Bharat

**Affiliations:** 1 Orthopaedic Surgery, King George's Medical University, Lucknow, IND

**Keywords:** dual-energy x-ray absorptiometry (dexa), speed of sound (sos), bone mineral density (bmd), osteoporosis, qus (quantitative ultrasonography)

## Abstract

Introduction

Bone mineral density (BMD) is an essential indicator for diagnosing osteoporosis and evaluating the success of osteoporotic treatment. Dual-energy X-ray absorptiometry (DEXA), quantitative ultrasonography (QUS), and quantitative computed tomography (QCT) are frequently used for measuring BMD. The objective of the study was to evaluate the ability of QUS to screen for osteoporosis and bone density in postmenopausal women by calibrating it against DEXA.

Methods

This cross-sectional study was conducted at the Department of Orthopedics and Trauma Center of the tertiary care center, Lucknow. A total of 90 patients visited this department from August 2017 to July 2018 for the present study. BMD in the same patient was evaluated by using DEXA and ultrasonography methods. Data were entered in Microsoft Excel and analyzed by using SPSS Software.

Results

According to linear regression analysis, T neck was found statistically significant with T QUS (p<0.001) and z QUS (p<0.001). T lumbar and T wrist were found statistically significant with T QUS (p<0.001) but not with z QUS (p>0.001). Z neck was found statistically significant with T QUS (p<0.001) and z QUS (p<0.001). Z lumbar was found statistically significant with T QUS (p<0.001) but not with z QUS (p>0.005). Z wrist was not found statistically significant with T QUS (p>0.005) or with z QUS (p>0.005).

Conclusion

In the present study, we found that QUS can be used as a screening tool for detecting osteoporosis by measuring BMD in contrast to DEXA. QUS also can be used to predict the DEXA values for osteoporosis and to detect osteoporosis.

## Introduction

Osteoporosis is a bone disease characterized by decreased bone mass and microarchitectural deterioration of bones, leading to enhanced fragility and fracture risk (WHO, 1994) [1]. Bone mineral density (BMD) decreases with age. Osteoporosis also causes an abnormally low bone mineral content, leading to bone fractures [2]. Assessment of BMD is essential in diagnosing osteoporosis and for follow-up therapy for osteoporosis [3-4]. Dual-energy X-ray absorptiometry (DEXA), quantitative ultrasonography (QUS), and quantitative computed tomography (QCT) are used to measure BMD. Various research has shown that BMD alone is insufficient in predicting the possibility of osteoporotic fracture. Various other factors, like the microstructure of bone and loading distribution over bone, also have a significant effect [3-7]. Therefore it is important to measure the changes in the trabecular microarchitecture [8]. DEXA is the gold standard. However, QUS is economical and accessible right up to survey level in a community setting and non-radiating compared to DEXA. The objective of the study was to evaluate the ability of QUS as a screening tool for osteoporosis and bone density by calibrating it against DEXA in Asian Indian postmenopausal women.

## Materials and methods

This cross-sectional study was conducted at a tertiary care center from August 2018 to July 2019. Ethical approval was taken from the Institutional Ethics Committee of King George's Medical University, U.P (approval #89ECM IIA/P12). Ninety postmenopausal women between the ages of 45 and 65 visited the department and were included in the present study. Exclusion criteria were patients with illnesses that can affect bone integrity, like endocrine diseases, Paget's disease, malignancy, renal failure or rheumatoid arthritis, hyperthyroidism, and prolonged immobilization. Complete medical history and physical examination were done for all patients. BMD was evaluated by using DEXA and ultrasonography methods in the same patients. T-score and z-score were calculated and compared to find an association between the two methods of evaluating BMD to diagnose osteoporosis in postmenopausal women. Data were entered in Excel and analyzed by using SPSS Software.

## Results

In the present study, the mean age of the study population was 55.82±6.80 years. Study participants had a median BMI of 25.40 with a range of 17.31-39.27, a mean height of 151.97±9.39 cm, and a mean weight of 60.05±11.46 kg. The most frequent comorbidity was back pain, found in 61.11% of cases, while a history of wrist fracture was found in 16.67% of cases, and a history of neck fracture was also seen in one case. Other symptoms were seen in 35.56% of cases. Among the DEXA readings, for neck and wrist, the median z-score was smaller than the T score, while for the lumbar, the median z and T scores were equal. In general, the z-score was smaller than the T score for DEXA and ultrasonography (Tables 1-2).

**Table 1:** Linear regression analysis showing relationship between T scores of DEXA and QUS. DEXA: Dual-energy X-ray absorptiometry; QUS: Quantitative ultrasonography; BMD: Bone mineral density.

Outcome variable	Independent variables	B	SE	T-value	P-value	Model fitness
T neck	(Constant)	-0.420	0.172	-2.435	0.017	70.50%
	T QUS	0.603	0.074	8.146	<0.001>	
	z QUS	0.287	0.056	5.126	<0.001>	
T lumbar	(Constant)	2.109	0.531	3.969	<0.001>	45.40%
	T QUS	1.592	0.228	6.980	<0.001>	
	z QUS	0.003	0.172	0.016	0.987	
T wrist	(Constant)	-1.002	0.288	-3.481	<0.001>	50.70%
	T QUS	0.848	0.124	6.860	<0.001>	
	z QUS	0.136	0.093	1.455	0.149	
T BMD	(Constant)	0.229	0.307	0.747	0.457	55.70%
	T QUS	1.014	0.132	7.704	<0.001>	
	z QUS	0.142	0.099	1.425	0.158	

**Table 2:** Linear regression analysis showing relationship between z scores of DEXA and QUS. DEXA: Dual-energy X-ray absorptiometry; QUS: Quantitative ultrasonography; BMD: Bone mineral density.

Outcome variable	Independent variables	B	SE	T-value	P-value	Model fitness
z neck	(Constant)	0.024	0.120	0.202	0.840	71.20%
	T QUS	0.370	0.051	7.185	<0.001>	
	z QUS	0.249	0.039	6.404	<0.001>	
z lumbar	(Constant)	2.470	0.407	6.064	<0.001>	45.10%
	T QUS	1.238	0.175	7.076	<0.001>	
	z QUS	-0.030	0.132	-0.225	0.822	
z wrist	(Constant)	-1.725	0.262	-6.585	<0.001>	16.60%
	T QUS	0.319	0.112	2.840	0.006	
	z QUS	0.075	0.085	0.880	0.382	
z BMD	(Constant)	0.257	0.211	1.217	0.227	52.10%
	T QUS	0.642	0.091	7.094	<0.001>	
	z QUS	0.098	0.068	1.432	0.156	

According to linear regression analysis, T neck was found to be highly correlated with T QUS (p<0.001) and z QUS (p<0.001), and the regression equation for predicting T neck using ultrasonography scores is given by T neck = -0.420 + 0.603×T QUS + 0.287×z QUS. T lumbar was found to be highly correlated with T QUS (p<0.001) but not with z QUS (p=0.987), and the regression equation for predicting T lumbar using ultrasonography scores is given by T lumbar = 2.109 + 1.592×T QUS + 0.003×z QUS. T wrist was found to be highly correlated with T QUS (p<0.001) but not with z QUS (p=0.149), and the regression equation for predicting T wrist using ultrasonography scores is given by T wrist = -1.002 + 0.848×T QUS + 0.136×z QUS. T BMD was found to be highly correlated with T QUS (p<0.001) but not with z QUS (p=0.158), and the regression equation for predicting T BMD using ultrasonography scores is given by T BMD = 0.229 + 1.014×T QUS + 0.142×z QUS.

According to linear regression analysis, z neck was found to be highly correlated with T QUS (p<0.001) and z QUS (p<0.001), and the regression equation for predicting z neck using ultrasonography scores is given by z neck = 0.024 + 0.370×T QUS + 0.249×z QUS. z lumbar was found to be highly correlated with T QUS (p<0.001) but not with z QUS (p=0.822), and the regression equation for predicting z lumbar using ultrasonography scores is given by z lumbar = 2.470 + 1.238×T QUS - 0.030×z QUS. z wrist was found to be highly correlated with T QUS (p=0.006) but not with z QUS (p=0.382), and the regression equation for predicting z wrist using ultrasonography scores is given by z wrist = -1.725 + 0.319×T QUS + 0.075×z QUS. z BMD was found to be highly correlated with T QUS (p<0.001) but not with z QUS (p=0.156), and the regression equation for predicting z BMD using ultrasonography scores is given by z wrist = 0.257 + 0.642×T QUS + 0.098×z QUS. Comparing the observed T scores with predicted sonography scores found that the average predicted value of T neck is 2.35±0.89, which almost matches the observed average value of 2.35±1.13. The unpaired t-test showed no significant difference between observed and predicted values (p=0.990). There is also a high value of correlation (r=0.784) between observed and predicted values. The average predicted value of T wrist is 3.36±1.46, which almost matches the observed average value of 3.36±1.03 and was found statistically insignificant (p=0.998). The average predicted value of T lumbar is 1.98±2.56, which almost matches the observed average value of 1.97±1.73, which was found statistically insignificant (p=0.995) (Table 3).

**Table 3:** Comparison of observed T-scores with predicted T-score of ultrasonography.

Score		Observed	Predicted	Difference	T-value	P-value	Correlation (r)
T neck	Mean	-2.35	-2.35	0.00	0.1192	0.990	0.784
	SD	1.13	0.89	0.70			
T wrist	Mean	-3.36	-3.36	0.00	0.1541	0.998	0.704
	SD	1.46	1.03	1.04			
T lumbar	Mean	-1.98	-1.97	0.00	0.2701	0.995	0.674
	SD	2.56	1.73	1.89			

The maximum sensitivity to detect osteoporosis using T-scores was found for T-neck with sensitivity = 86.36%, specificity = 86.76%, positive predictive value (PPV) = 67.86%, and negative predictive value (NPV) = 95.16%. The most specific test to detect osteoporosis using T- scores was T-wrist with sensitivity = 50.0%, specificity = 90.48%, PPV = 85.71%, and NPV = 61.29%. The maximum sensitivity to detect osteoporosis using z-scores was found for z-neck with sensitivity = 100.0%, specificity = 93.10%, PPV = 33.33%, and NPV = 100.0%. The most specific test to detect osteoporosis using z-scores was z-wrist with sensitivity = 29.63%, specificity = 98.41%, PPV = 88.89%, and NPV = 76.54%.

## Discussion

Results from this study demonstrate a significant correlation between QUS and BMD sites. Larijani B et al. (2005) [9] studied 420 women and determined BMD categories in the lumbar spine and left femur. The sensitivity of QUS at the lumbar spine was 83.9%, and specificity was 51%, respectively, and at the femoral neck, 84% and 50%, respectively. In the study by Sözen T et al. [10], the mean specificity of QUS vs. DEXA was 45%, and the mean sensitivity was 79% over a seven-year period. In conclusion, DEXA and QUS were well correlated, and QUS can prove helpful in setups that do not have access to DEXA. The specificity was low, but the sensitivity was high, implicating that checking that a facility of DEXA is available is recommended before starting the treatment for osteoporosis. In Cetin A et al. study [11], correlations of both right and left speed of sound (SOS) and broadband ultrasonic attenuation (BUA) with the spine and femoral neck BMD were moderately correlated (r=0.343-0.539, P < 0.001). There was also a reasonable correlation between DEXA and QUS T-scores (r=0.364-0.510, P < 0.001). QUS had a sensitivity of 21% and a specificity of 95% for diagnosing osteoporosis. The linear regression results between QUS and DEXA were used to define an estimated confidence range of 95% for BMD. Correlation coefficients between the DEXA and QUS ranged from 0.26 to 0.63. Therefore, they conclude that QUS is unsuited for screening early postmenopausal women for low peripheral axial BMD. However, it may have a role as an independent predictor of fracture by measuring skeletal properties in addition to bone density. Clearly, Z-scores and T-scores obtained from calcaneum are sensitive to changes in bone mass and should be used as an early screening tool for detecting changes in bone mass. The DEXA values can be predicted with the help of QUS values by the study-generated nomograms [12-13]. Therefore, the results of our study confirm QUS to be a good screening tool with sensitivity for T scores >50% (i.e., a minimum for T wrist is 50% and maximum for T neck is 86.36%) and for Z scores >29.63% (i.e., a minimum for Z wrist is 29.63% and maximum for z neck is 100%) in identifying patients of osteoporosis. It has the capability to differentiate between young, pre and postmenopausal females. It can be an alternative to traditional radiation-based technologies in a community-based setting where heavy infrastructure is unavailable. One limitation of this study was the small sample size, which may affect the generalizability of the findings. In addition, the study was conducted in a single geographic location, which may have limited the diversity of the sample. Finally, the study was cross-sectional in nature which precluded any casual interference. 

## Conclusions

In our study, we found that the QUS can be used as a screening tool for detecting osteopenia and osteoporosis by being a safe (non-radiating), accessible, and economical alternative to DEXA. QUS can be an alternative option to measure BMD during surveys in contrast to DEXA. QUS can also be used to predict the DEXA values with maximum sensitivity to detect osteoporosis. For osteoporosis, the T score and z score of the neck of the femur on QUS is the best score to predict the T score and z score of the neck of the femur on DEXA.
